# Preparation and Characterization of Chitosan/TiO_2_ Composite Membranes as Adsorbent Materials for Water Purification

**DOI:** 10.3390/membranes12080804

**Published:** 2022-08-20

**Authors:** Angela Spoială, Cornelia-Ioana Ilie, Georgiana Dolete, Alexa-Maria Croitoru, Vasile-Adrian Surdu, Roxana-Doina Trușcă, Ludmila Motelica, Ovidiu-Cristian Oprea, Denisa Ficai, Anton Ficai, Ecaterina Andronescu, Lia-Mara Dițu

**Affiliations:** 1 Department of Science and Engineering of Oxide Materials and Nanomaterials, Faculty of Chemical Engineering and Biotechnologies, University Politehnica of Bucharest, 1-7 Gh Polizu Street, 011061 Bucharest, Romania; 2 National Centre of Micro and Nanomaterials, Faculty of Chemical Engineering and Biotechnologies, University Politehnica of Bucharest, Spl. Indendentei 313, 060042 Bucharest, Romania; 3 National Center for Scientific Research for Food Safety, University Politehnica of Bucharest, Spl. Indendentei 313, 060042 Bucharest, Romania; 4 Department of Inorganic Chemistry, Physical Chemistry and Electrochemistry, Faculty of Chemical Engineering and Biotechnologies, University Politehnica of Bucharest, 1-7 Gh Polizu Street, 050054 Bucharest, Romania; 5 Academy of Romanian Scientists, 3 Ilfov Street, 050045 Bucharest, Romania; 6 Faculty of Biology, University of Bucharest, 1-3 Aleea Portocalelor, 060101 Bucharest, Romania

**Keywords:** TiO_2_, chitosan, composite membranes, adsorbent, heavy metal ions, water purification, visible light photocatalysis, antibacterial activity

## Abstract

As it is used in all aspects of human life, water has become more and more polluted. For the past few decades, researchers and scientists have focused on developing innovative composite adsorbent membranes for water purification. The purpose of this research was to synthesize a novel composite adsorbent membrane for the removal of toxic pollutants (namely heavy metals, antibiotics and microorganisms). The as-synthesized chitosan/TiO_2_ composite membranes were successfully prepared through a simple casting method. The TiO_2_ nanoparticle concentration from the composite membranes was kept low, at 1% and 5%, in order not to block the functional groups of chitosan, which are responsible for the adsorption of metal ions. Nevertheless, the concentration of TiO_2_ must be high enough to bestow good photocatalytic and antimicrobial activities. The synthesized composite membranes were characterized by Fourier transform infrared spectroscopy (FTIR), X-ray diffraction (XRD), scanning electron microscopy (SEM), thermogravimetric analysis (TGA) and swelling capacity. The antibacterial activity was determined against four strains, *Escherichia coli, Citrobacter* spp., *Enterococcus faecalis* and *Staphylococcus aureus*. For the Gram-negative strains, a reduction of more than 5 units log CFU/mL was obtained. The adsorption capacity for heavy metal ions was maximum for the chitosan/TiO_2_ 1% composite membrane, the retention values being 297 mg/g for Pb^2+^ and 315 mg/g for Cd^2+^ ions. These values were higher for the chitosan/TiO_2_ 1% than for chitosan/TiO_2_ 5%, indicating that a high content of TiO_2_ can be one of the reasons for modest results reported previously in the literature. The photocatalytic degradation of a five-antibiotic mixture led to removal efficiencies of over 98% for tetracycline and meropenem, while for vancomycin and erythromycin the efficiencies were 86% and 88%, respectively. These values indicate that the chitosan/TiO_2_ composite membranes exhibit excellent photocatalytic activity under visible light irradiation. The obtained composite membranes can be used for complex water purification processes (removal of heavy metal ions, antibiotics and microorganisms).

## 1. Introduction

In the last several decades, water pollution has become a paramount concern that equally affects the environmental system and humans [1,2]. Because of various pollutants (antibiotics [3], pesticides [4], heavy metals [5], dyes [6], fluoride [7], chlorinated hydrocarbons [8], etc.) present in wastewaters, there is a need to develop innovative membranes to help mitigate this problem [9]. Therefore, removing these toxic pollutants from wastewater is of primordial importance [10,11]. Produced water is a by-product of the oil and gas industries that needs to be treated before it can be used in agriculture or recirculated towards other industries. Composite membrane solutions based on the zeolitic imidazole framework and polyacrylonitrile are reported in the literature [12]. The presence of pollutants in water systems significantly affects the surroundings and human health [2,13,14,15]. Researchers and scientists are trying to overcome this water issue by developing new alternative materials with excellent adsorbent properties [16,17,18]. Numerous traditional methods are used to remove these toxic pollutants [19], such as reduction, co-precipitation, ultrafiltration, sedimentation, photocatalysis, distillation and adsorption methods [2,13]. Mixed matrix membranes (MMMs) consisting of a polymeric matrix and an inorganic filler have the potential to achieve high performance in water depollution by mixing the capabilities of the organic polymer (such as adsorption of heavy metals) with those of the inorganic nanoparticles (such as antimicrobial action leading to fouling mitigation) [7,20]. The membranes are not only used for removal of pollutants. In the biotechnology, food and pharmaceutical industries, ultrafiltration membranes, such as polyethersulfone hollow fibers, are used for the efficient separation of biomolecules [21,22].

Among materials proposed for water purification, chitosan-based ones present a great interest because of their free amino and hydroxyl groups, good biocompatibility, biodegradability, nontoxicity, reactivity, hydrophilicity and cost effectiveness [2,11,23,24]. The fact that chitosan presents hydroxyl and amine groups on its surface is the reason for its wide use in heavy metal removal from wastewaters [25,26,27,28,29,30,31]. However, it does have some inconveniences regarding its low stability, thermo-mechanical properties and porosity [32]. Scientists have developed chitosan-based adsorbent membranes to overcome these difficulties through diverse methods [33,34,35,36,37]. Functionalization with glyoxal, glutaraldehyde or epichlorohydrin can reinforce the structure of chitosan through cross-linking, enhancing its mechanical properties. Studies have reported that through cross-linking, the adsorption capacity of chitosan membranes has improved substantially [38,39,40,41]. Various chitosan-based membranes have been developed for the removal of pollutants from wastewaters [42]. Many studies have demonstrated that chitosan exhibits some weak antibacterial and antioxidant properties, which can also help in the water purification processes [43,44,45].

Titanium dioxide (TiO_2_) is an industrial pigment, disinfectant agent and photocatalyst, having excellent thermo-stability and low toxicity. TiO_2_ has presented great performance in environmental applications. In addition to photocatalytic and antibacterial properties, TiO_2_ presented potential in water treatment applications due to the induced porosity to the composite membrane [23,46,47,48,49]. Gonzalez-Calderon et al. [50] and Li et al. [51] found that TiO_2_ incorporation in chitosan membranes improves the mechanical, physicochemical, thermal and UV protection of the composite membrane, and also that TiO_2_ substantially enhances the antimicrobial activity against *E. coli* and *S. aureus*. However, it has been proven that a considerable concentration of TiO_2_ on the biopolymer matrix could cause the aggregation of inorganic nanoparticles onto the surface of the composite, thus affecting the mechanical properties [52]. Razzaz et al. [53] reported a composite chitosan/TiO_2_ membrane that demonstrated high adsorption capacity for removing Cu(II) and Pb(II) ions from water systems. Elsewhere, Samadi et al. [54] presented a novel Cu–TiO_2_/chitosan hybrid thin film used to remove heavy metals from aquatic media. The study of Chien et al. [55] on chitosan/TiO_2_ composites confirmed the adsorption capacity of this solution, but the capacity was not impressive. In another study, the authors developed a novel magnetic EDTA/chitosan/TiO_2_ (MECT) nanocomposite to remove Cd(II) metal ions and phenol as hazardous materials from aqueous solutions [56]. In fact, many literature studies report adsorption values under 100 mg/g, mainly because the composite ratio of chitosan to TiO_2_ is close to 1:1 or even more as it is in [55]. Such high TiO_2_ content in the composite membrane will adversely affect the capacity of the chitosan to further bind the heavy metal ions, but in theory should improve the photocatalytic activity of the membrane. Using a lower TiO_2_ concentration for the composite membrane should allow a better adsorption capacity vs. heavy metal ions, with only a small diminishing of the photocatalytic activity.

Most of the literature reports regarding the photocatalytic activity of TiO_2_ are based on UV [57,58,59] or simulated sun light [60,61]. In theory, introducing intermediary electronic levels into the semiconductor’s band gap allows photocatalytic activity under visible light irradiation. Such intermediary electronic levels can be generated by doping or by obtaining a high surface defect density on the TiO_2_ nanoparticles during synthesis.

TiO_2_, like many other oxides with photocatalytic activity [62,63], generates reactive oxygen species (ROS), which ensures antimicrobial activity against various microorganisms. Chitosan also presents weak antimicrobial activity on its own [44]. Therefore, such a composite membrane should exhibit antimicrobial activity, but due to synergism a strong bactericidal action can arise.

Our aim was to develop novel chitosan/TiO_2_ adsorbent composite membranes, with low TiO_2_ content, for complex water purification. To our knowledge, this is the first time that this membrane is reported to be used as an adsorbent for heavy metal ions, as a photocatalytic system against an antibiotic mixture and as an antibacterial agent, accomplishing thus a complex water purification process. We used previously synthesized TiO_2_ through a simple sol-gel method [64] that yields impurity-free nanoparticles, but with high surface defect density, which allowed the use of visible light in photocatalysis. The chitosan/TiO_2_ composite membranes were prepared through a simple casting method and further characterized by FTIR, XRD, TG-DSC-FTIR and SEM. Swelling capacity, heavy metal ion adsorption capacity, photocatalytic degradation of a five-antibiotic mixture and antibacterial activity were also determined.

## 2. Materials and Methods

### 2.1. Experimental

Titanium isopropoxide, having ≥ 97.0% purity, was acquired from Sigma Aldrich (Merck, Burlington, MA, USA). Isopropanol (2-propanol) with 99.99% purity was obtained from Sigma Aldrich (Merck, Burlington, MA, USA). Nitric acid 65% was from Sigma Aldrich (Merck, Burlington, MA, USA). Chitosan (CS) (molecular weight 100.000–300.000, Acros Organics, Geel, Belgium), glacial acetic acid (AcA) (Chimreactiv, Bucharest, Romania) and sodium hydroxide ≥ 97.0% were from Sigma Aldrich (Merck, Burlington, MA, USA). Glutaraldehyde (GA) (50% in water) was purchased from Sigma Aldrich (Merck, Burlington, MA, USA) and distilled water was used. All chemicals used in the present study were of analytical grade without further purification.

The microbiological activity was performed using Nutrient Broth No. 2 and agar, acquired from Sigma-Aldrich (Darmstadt, Germany). All strains tested in this study were provided by the Microorganisms Collection of the Department of Microbiology, Faculty of Biology and Research Institute of the University of Bucharest.

TiO_2_ was synthesized using a simple sol-gel method as described in [64]. Briefly, titanium isopropoxide was added drop by drop into a solution containing isopropanol and distilled water to obtain titanium dioxide nanoparticles. The obtained solution was magnetically stirred on a hot plate at almost 80 °C. After 1 h of stirring, a solution containing concentrated nitric acid and distilled water was added to the previous solution and kept under constant stirring on a hot plate at 60 °C for 6 h until a white sol-gel appeared. The appearance of the white sol-gel indicates the formation of titanium dioxide nanoparticles. After the precipitate was washed several times for residual removal, it was heated in an electric oven at 300 °C for 2 h. The powders were then placed in a furnace at 550 °C for 5 h. The as-obtained TiO_2_ nanoparticles were further used to develop the composite membranes described below.

**Figure 1:**
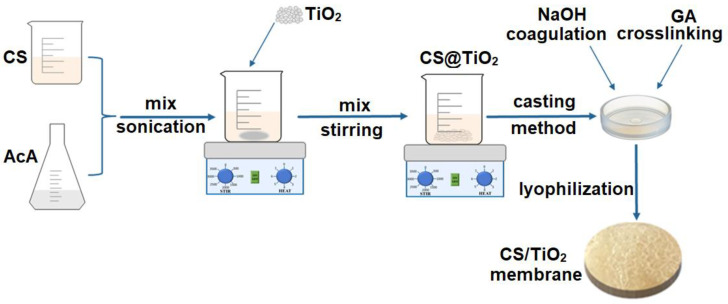
Schematic representation of the preparation of chitosan/TiO_2_ composite membranes (CS—chitosan; AcA—acetic acid; GA—glutaraldehyde).

Figure 1 illustrates a schematic chart of the preparation of chitosan/TiO_2_ composite membranes through a simple casting method. First, chitosan (2 g) was dissolved in a 1% acetic acid solution. Then, TiO_2_ was added to the as-obtained chitosan solution previously synthesized through the sol-gel method; afterwards, the mixture was magnetically stirred at room temperature for 24 h and then sonicated for 4 h at 35 °C to obtain a homogenous solution. For this experiment, two polymer solutions with 1% and 5% (*w/w*) TiO_2_ nanoparticles were obtained. The solutions were cast into Petri dishes and dried in an electric oven at 50 °C overnight. The obtained membranes were treated with NaOH solution for 24 h to coagulate the chitosan; afterwards, they were washed with distilled water to remove side products and excess NaOH. To cross-link the obtained membranes, they were placed in a diluted glutaraldehyde solution (200 mL, 2% *v/v*) for 24 h under magnetic stirring. This was followed by washing with distilled water to remove any remains from the glutaraldehyde. The synthesized composite membranes were further lyophilized and analyzed through proper techniques.

### 2.2. Characterization

The synthesized membranes were characterized by Fourier transform infrared spectroscopy (FTIR), X-ray diffraction (XRD), thermogravimetric analysis (TG) and differential scanning calorimetry (DSC) coupled with the FTIR analysis of the evolved gases and scanning electron microscopy (SEM).

The Fourier transform infrared spectroscopy (FTIR) measurements were performed using a Nicolet iS50R spectrometer (Thermo Fisher Scientific, Waltham, MA, USA). The spectra were recorded at room temperature using the attenuated total reflection (ATR) (Thermo Fisher Scientific, Waltham, MA, USA), with 32 scans between 4000 and 400 cm^−1^ at a resolution of 4 cm^−1^, with the scanning time being 47 s.

FTIR 2D maps were recorded with a Nicolet iS50R FTIR microscope (Thermo Fisher Scientific Inc., Waltham, MA, USA), with a DTGS detector, in the wavenumber range 4000–600 cm^−1^. The 2D FTIR maps were used to obtain information about the spatial distribution of the components.

X-ray diffraction (XRD) experiments were carried out on a Panalytical Empyrean instrument (Malvern Panalytical, Malvern, UK) with Ni-filtered Cu radiation (λ = 0.15406 Å) equipped with a 1/4° fixed divergence slit and a 1/2° anti-scatter slit on the incidence beam side, and a 1/2° anti-scatter slit mounted on a PIXCel3D detector (Malvern Panalytical, Malvern, UK) on the diffracted beam side. Data reduction and analysis of the patterns were performed in HighScore Plus 3.0.e software (Malvern Panalytical, Malvern, UK) coupled with the ICDD PDF4 + 2021 database.

The thermogravimetric analysis (TG-DSC) was performed with an STA 449 F3 Jupiter apparatus from Netzsch (Selb, Germany) coupled with an FTIR Tensor 27 from Bruker. Approximately 10 mg of dry powder was placed in an open alumina crucible and heated up to 900 °C with a 10 °C min^−1^ rate under a flow of 50 mL min^−1^ of dried air. As a reference, an empty alumina crucible was used.

The electron microscopy images (SEM) were obtained using a Quanta Inspect F50 (FEI Company, Eindhoven, The Netherlands) equipped with a field emission gun (FEG) with a 1.2 nm resolution and an energy dispersive X-ray spectrometer (EDS) with an MnK resolution of 133 eV Kα.

The heavy metal ion retention capacity of the chitosan/TiO_2_ composite membranes was evaluated by inductively coupled plasma–mass spectrometry (ICP-MS). Before ICP-MS analysis, samples of each membrane (~1 g) were exposed for 2 h to 50 mL 1% and 5% lead nitrate solutions and 1% and 5% cadmium nitrate solutions (both from Sigma Aldrich, Darmstadt, Germany). Nitrate solutions have been used as sources of heavy metals found in polluting aqueous media, having a substantial negative impact on the environment, including animal and human health. After exposure to the previously mentioned solutions, our membranes were removed from the Pb(NO_3_)_2_ and Cd(NO_3_)_2_ solutions, washed with distilled water and placed in the oven at 105 °C until complete drying.

The retention of heavy metals on the composite membranes was evaluated considering cadmium and lead concentrations. Cadmium and lead concentrations were determined using an Agilent 8800 Triple Quadrupole ICP-MS (Agilent Technologies, Tokyo, Japan), equipped with an ASX500 autosampler, MicroMist concentric nebulizer, Peltier cooling spray-chamber (2 °C), 2.5 mm internal diameter torch, nickel sampler and skimmer cones. Operating conditions of ICP-MS included 1550 W RF power, 1 L/min carrier gas flow, 0.7 mL/min He flow and nebulizer pump set to 0.1 RPS. Before ICP-MS analysis, the dried membranes were subjected to digestion. Three samples of ~200 mg of each previously treated membrane were digested with 8 mL HNO_3_ in a microwave oven (Ethos UP, Milestone Inc., Sorisole, Italy), applying a dedicated program for high organic content matrices (200 °C heating, 1800 W microwave power) for 35 min. After cooling, the digestion samples were diluted with Milli-Q water up to 50 mL and filtered through 0.45 μm pore size syringe filters. To achieve the best sensibility, samples were diluted 100,000 times with ultrapure water. Before measurements, ICP-MS was tuned according to the manufacturer and calibrated with five calibration standards ranging from 0.1 to 5 µg/L Cd and Pb. Our results were verified by a certified reference material (SRM 1567b) subjected to the same preparation steps as the samples and calculating the percent recovery. Calibration curves of the two elements proved linear in the operating range of 0.1–5 μg/L, with correlation coefficients greater than 0.999. Cadmium recovery from the SRM 1567b sample was 98%, and lead recovery was 107%, considered acceptable in instrumental analysis. The final metal concentration reported for dry membrane mass was calculated using the following formula:

$$Metal\;final\;concentration\;\left(µg\cdot mg^{-1}\right)=\frac{C\times F_{d}\times V}{M}$$

where *C* is the raw concentration read by ICP-MS, *F_d_* is the dilution factor (100,000), *V* is the final digestion volume (0.05 L) and *M* is the dry sample mass (mg).

The photocatalytic activity of the chitosan/TiO_2_ membranes was determined against a solution with five antibiotics (50 ppb vancomycin, meropenem, tetracycline, clindamycin and erythromycin) by irradiation with a LOHUIS^®^ (Ulmi, Romania), commercially available, and a fluorescent lamp of 160 W/2900 lm (lumen), with a color temperature of 3200 K and color rendering index > 60, placed at 20 cm distance. The chitosan/TiO_2_ samples of the ~40.0 mg disk were inserted into a 50 mL solution of antibiotic mix. After irradiation, at defined time intervals, a sample of 1 mL was analyzed by LC-MS to quantify the quantity of the antibiotic. Three groups of parallel experiments were set up.

The antibiotic degradation by the tested membranes was evaluated with liquid chromatography–mass spectrometry (LC-MS). The LC-MS analyses were performed on Agilent Technologies 6540 UHD Accurate-Mass Q-TOF LC/MS equipped with a reversed-phase Zorbax Eclipse C18 (Agilent, 50 × 4.6 mm, 2.7 μm particle size). Mobile phases were 95% water (A) and 5% acetonitrile (B). Antibiotics were separated following a gradient program: initial conditions were 95% A, then the gradient was from 100% B to 5% B in 5 min, and finally, solvents were maintained at 95% A and 5% B for 4 min. The total run time was 14 min. The column temperature was 60 °C, and the flow rate was 0.15 mL/min. The sample injection volume was set at 5 μL. The mass spectrometer was a Q-TOF system with a Dual ESI ion source operated in positive ionization mode. The operating parameters were ion spray voltage 5300 V, drying gas, 7 L/min, nebulizer gas 21 psig and probe temperature 300 °C. The acquisition rate was 1.1 spectra/s, 909.1 ms/spectrum for each compound. 

The swelling capacity was determined by immersing square pieces 2 cm × 2 cm in 200 mL water. Each piece was weighed before starting the experiment and at fixed time intervals: 0.25, 0.5, 1, 2, 4, 6, 12 and 24 h. The experiments were performed in triplicate. The following formula calculated the water uptake capacity of the samples:

***Water retention (%) =***$\frac{M_{h,t}-M_{i}}{M_{i}}\times 100$, where *M_i_* is the initial weight and *M_h,t_* is the weight after immersion in water.

The antibacterial assessments of the composite membranes were made to evaluate their potential use in water purification applications. The anti-adherent capacity of the membranes obtained in this study was conducted by determining the colony-forming units/mL values (CFU/mL). The antibacterial activity was evaluated against the *Staphylococcus aureus* MRSA 5578, *Enterococcus faecalis* VRE 2566, *Escherichia coli* ATCC 25922 and *Citrobacter* sp 2021. The samples (1 cm/1 cm) were previously sterilized under UV radiation for 30 min on each side in order to eliminate any possible contamination. To confirm the sterility of the tested samples before the antibacterial assay, each type of membrane was maintained in nutrient broth media for 24 h at 37 °C. The clarity of the broth media confirmed the sterility of the samples.

Bacterial cell suspensions (1.5 × 10^8^ CFU/mL) were made in a sterile physiological buffer from fresh cultures (18–24 h). The anti-adherent capacity of the samples was performed using the method described in the previous studies [65,66,67] and according to the CLSI standard [68]. The negative control was considered the sterile media, and the positive control (C+) was the broth media inoculated with microbial suspensions. The CFU/mL values were expressed as the average of the total number of colonies × 1/D (D = decimal dilution, for which the number of total colonies was to be determined). The assays were performed in three independent experiments.

Antibacterial assessments were performed in triplicate and were analyzed using GraphPad Prism 9 by GraphPad Software, San Diego, CA, USA. We compared the ability of selected strains to adhere to the surface of the membranes using analysis of variance (ANOVA) and Dunnett’s multiple comparisons test. A *p*-value < 0.05 is considered statistically significant.

## 3. Results and Discussion

### 3.1. Fourier Transform Infrared Spectroscopy (FTIR) Analysis

**Figure 2:**
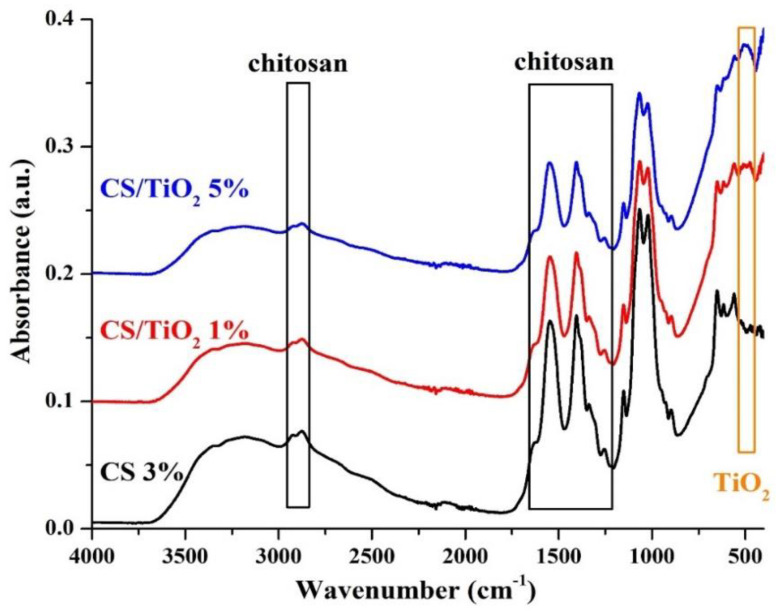
FTIR analysis of chitosan and chitosan/TiO_2_ composite membranes.

FTIR analysis is a valuable technique for determining and detecting chemical bonds inside a material. The FTIR spectra of chitosan and chitosan/TiO_2_ composite membranes are presented in Figure 2, and the assignment of the relevant peaks is made in Table 1. The chitosan exhibits several peaks corresponding to the functional groups: -OH and –NH_2_ in the 3100–3500 cm^−1^ region, the C-H stretching vibration at 2875–2925 cm^−1^ (symmetric and asymmetric), amide band I, II and III in the region 1630–1300 cm^−1^ and asymmetric vibration of a C-O-C group at ~1150 cm^−1^ [69]. These peaks are observable for all samples.

Additionally, the presence of TiO_2_ produces a double peak at ~500 cm^−1^ due to the Ti-O stretching vibration (observable only in chitosan/TiO_2_ samples). Comparison between the chitosan and chitosan/TiO_2_ composite membranes indicates their interactions; this interaction is represented by the formation of Ti-O-Ti stretching bonds at wavelengths ranging from 476 cm^−1^ to 508 cm^−1^ [70,71].

**Figure 3:**
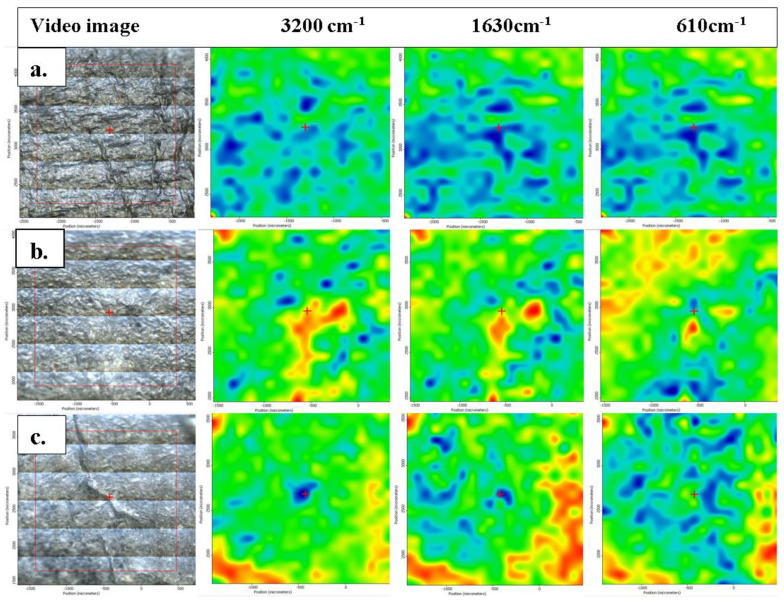
FTIR microscopy images recorded of the samples: (**a**) CS 3%; composite membranes (**b**) chitosan/TiO_2_ 1% and (**c**) chitosan/TiO_2_ 5%;the red indicates the zones with high absorbance, while the blue corresponds to the zones with low absorbance.

The spatial distribution of TiO_2_ nanoparticles inside the chitosan matrix can be investigated by FTIR microscopy. The microscope records the full FTIR spectra for each point, creating a 2D map that can be displayed for any chosen wavelength. The FTIR maps recorded at 3200, 1630 and 610 cm^−1^ for the chitosan control and the composite membranes (chitosan/TiO_2_ 1% and chitosan/TiO_2_ 5%) are presented in Figure 3. Interactions between various components of a composite sample will lead to zonal modification of absorption at certain characteristic wavelengths. When 2D FTIR maps present distinct zones at different characteristic wavelengths, it can be interpreted as a localized interaction proof between components. Mono-component or highly homogeneous mixtures will yield similar 2D maps for different wavelengths, and for inhomogeneous samples some differences between maps can be visualized. The membranes present a high degree of homogeneity, with some agglomerated clusters and surface defects. As expected, the FTIR maps recorded for the simple chitosan membrane are consistent at all three wavenumbers. In the case of the composite membranes, chitosan/TiO_2_, the maps recorded at 3200 and 1630 cm^−1^ are similar, with only minor differences. Nevertheless, the maps recorded at 610 cm^−1^ present noticeable differences for the composite membranes compared with the 3200 and 1630 cm^−1^ maps, indicating the presence of TiO_2_ nanoparticles that interact with the chitosan matrix and induce shifts in the position of the absorption peaks. Based on the information provided by FTIR microscopy, the spatial distribution of the TiO_2_ nanoparticles in the composite membranes can be considered good, with occasional agglomerations on the micrometer level.

### 3.2. X-ray Diffraction (XRD) Analysis

**Figure 4:**
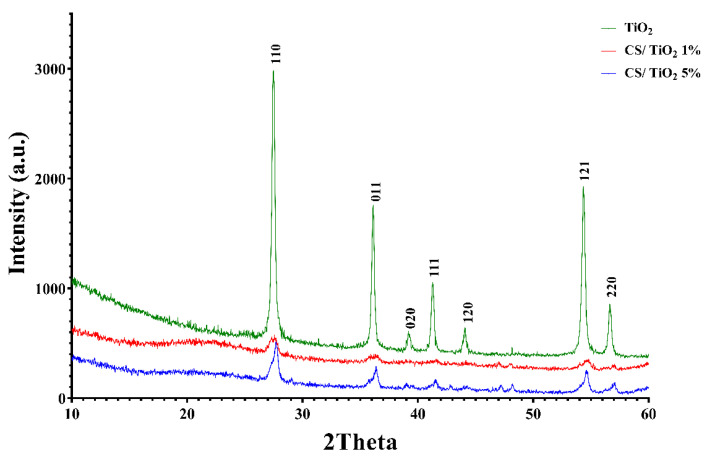
XRD analysis of TiO_2_ and chitosan/TiO_2_ composite membranes.

An XRD analysis was carried out (Figure 4) to reveal the crystalline structure of the synthesized chitosan and chitosan/TiO_2_ composite membranes.

The Scherrer equation was used to determine the crystallite size (~31.36 nm) of TiO_2_ in rutile form. The XRD diagram confirms the presence of common peaks of TiO_2_ (crystalline) and a broad phase ranging from 10 to 25, corresponding to chitosan (slightly amorphous) [72].

### 3.3. Thermal (TG-DSC) Analysis

**Figure 5:**
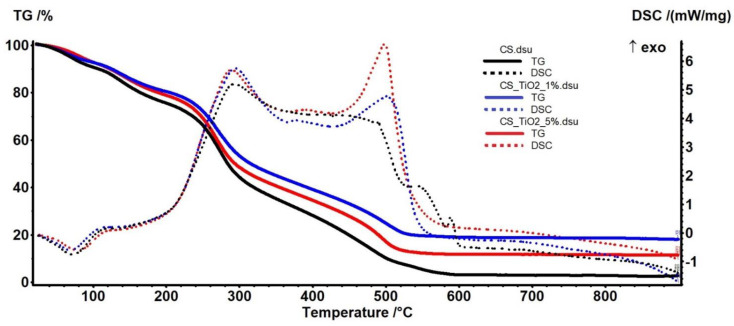
TG-DSC curves of chitosan and chitosan/TiO_2_ composite membranes.

The thermal analysis results are presented in Figure 5 for chitosan and chitosan/TiO_2_ composite membranes. The comparison between the samples can be made based on the data from Table 2. The samples are losing residual water molecules up to 105 °C (~7–10%), and a weak endothermic effect accompanies the process on the DSC curve [73].

The chitosan control sample exhibits a higher mass loss, which is expected as there is no inorganic part in it. The degradative process starts after 150 °C, when chitosan molecular chains break free and residual acetic acid is eliminated (~11–14%).

**Figure 6:**
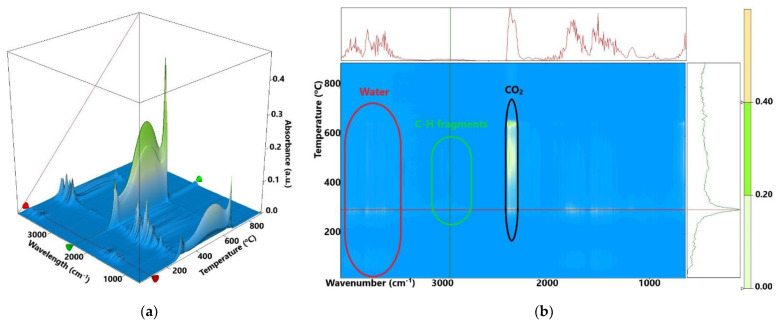
The evolved gases FTIR 3D diagram for the CS/TiO_2_ 5% sample (**a**) and its 2D projection with assigned identification/temperature zones (**b**); the yellow-green indicates the zones with high absorbance, while the light shade of blue corresponds to the zones with low absorbance.

After 200 °C up to 370 °C, the samples lose ~40% of their mass in a complex degradative-oxidative process. The polysaccharide chains are broken, and the smaller fragments are oxidized. This process is accompanied by a strong, sizeable exothermic effect, with a maximum at ~290 °C generated by the oxidation of the organic fragments. The FTIR spectra of the evolved gases (Figure 6) permit identification of water, CO_2_ and hydrocarbon fragments (such as acetic acid) in this temperature interval. The larger organic fragments are slowly oxidized, and residual carbonaceous mass is burned after 370 °C, the corresponding effects on the DSC curve being exothermic (~500 °C).

As expected, the residual mass is higher as the proportion of inorganic TiO_2_ in the sample increases up to 5%.

The 3D FTIR plot (Figure 6a) presents the evolution of the FTIR spectrum vs. temperature. By projecting this map in 2D space (wavenumber vs. temperature—like a topographical map) we can easily identify the components and temperature intervals when they are eliminated from the sample (Figure 6b). The FTIR spectra recorded for the evolved gases indicate the presence of water and carbon dioxide molecules, but also some traces of carbon monoxide. Hydrocarbon fragments can be identified starting from 250 °C.

### 3.4. Scanning Electron Microscopy (SEM) Characterization

The SEM analysis gives us information regarding the surface morphology and possible fractures that could appear inside the membranes. For the SEM characterization, the samples were coated with a thin layer of gold before using them for analysis.

**Figure 7:**
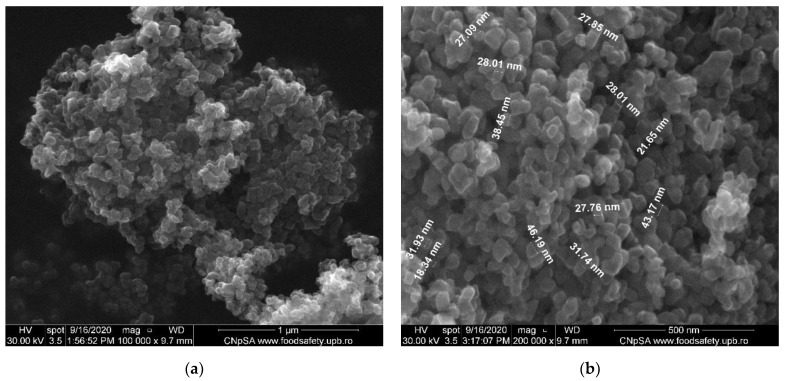
SEM images of TiO_2_ nanoparticles: (**a**) 100.000× magnification, (**b**) 200.000× magnification.

The as-synthesized TiO_2_ nanoparticles were examined by SEM (shown in Figure 7). They have polyhedral shapes and a tendency to form agglomerates. The individual TiO_2_ nanoparticles presented uniformity in size (average of 30.8 nm) and shape. Correlating the values from SEM with those calculated by the Scherrer equation from XRD, we can conclude that each nanoparticle contains a single crystallite grain.

**Figure 8:**
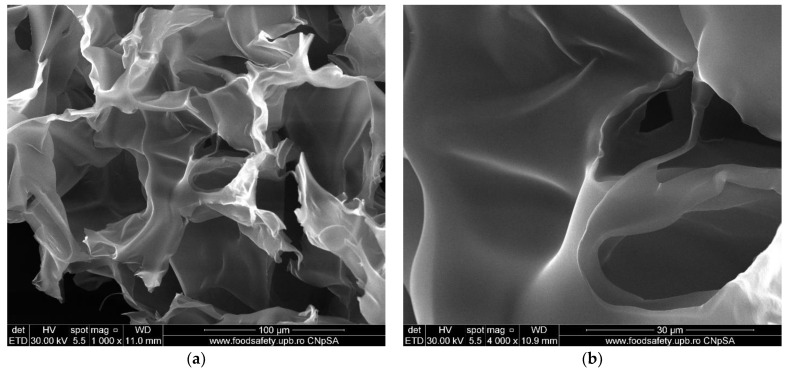
SEM images of simple chitosan CS membrane: (**a**) 1000× magnification, (**b**) 4000× magnification.

The morphology of the simple chitosan (CS) membrane is presented in Figure 8. The membrane is highly porous, with labyrinthic pores. Such pores are the result of the coagulation and cross-linking processes when large flakes become fused under various shapes. The lyophilization process also plays an important role in forming the smaller pores by forcing the water out of the structure. The result is a sponge-like appearance of the membrane, similar to that in other literature reports [44,74,75].

**Figure 9:**
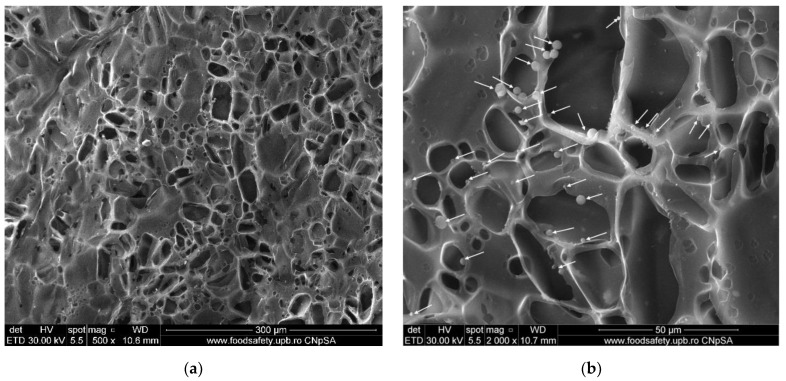

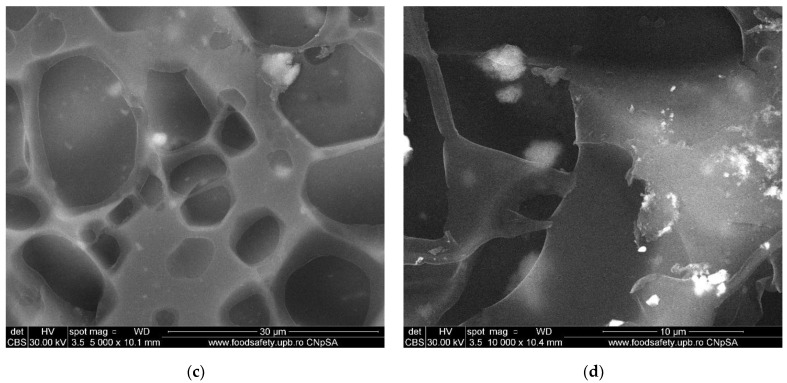
SEM images of chitosan/TiO_2_ 1%: (**a**) 500× magnification, (**b**) 2000× magnification, (**c**) 5000× magnification and (**d**) 10,000× magnification.

From the analysis of SEM micrographs, one may observe that the as-prepared composite membranes have a different structure, which may be caused by the higher concentration of nanoparticles within the chitosan matrix (for chitosan/TiO_2_ 5%). In the first case, the chitosan/TiO_2_ 1%, the well-interconnected and homogenous porous microstructure generated during the lyophilization process can be observed (Figure 9a). This porous structure is decorated with TiO_2_ nanoparticle agglomerations (marked with arrows in Figure 9b). Higher magnifications (Figure 9c,d) reveal the stand alone nature of the pores (with no interconnections). The pores are smaller than those of the simple chitosan membrane, but still numerous and large enough, due to the presence of the TiO_2_ nanoparticles that act as cross-linking points, stitching the chitosan polymer chains together. Multiple nanoparticle agglomerates are visible on the pore surfaces (Figure 9d). The pore formation within the membranes might lead to enhanced adsorption capacity vs. various pollutants presented in wastewaters, which is significant in developing adsorbent membranes for water purification applications.

**Figure 10:**
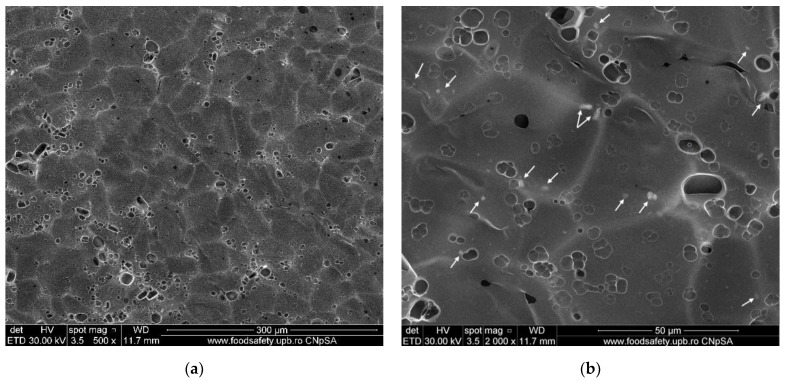

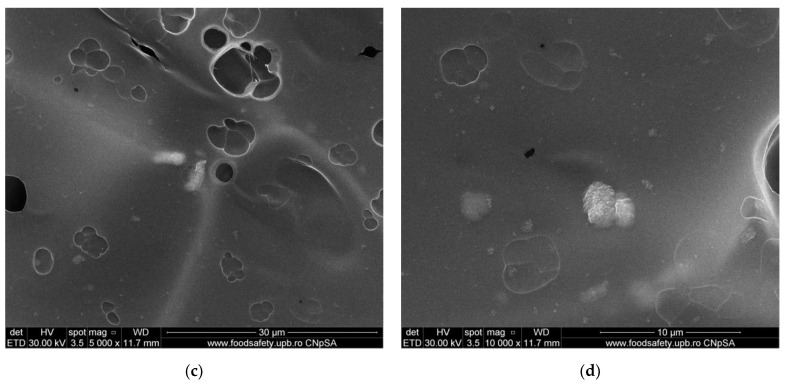
SEM images of chitosan/TiO_2_ 5%: (**a**) 500× magnification, (**b**) 2000× magnification, (**c**) 5000× magnification and (**d**) 10,000× magnification.

The chitosan/TiO_2_ 5% has a smoother surface (Figure 10a), on which numerous smaller pores are visible (smaller and less numerous when compared with CS or chitosan/TiO_2_ 1% membranes). Adding a higher concentration of TiO_2_ nanoparticles has reduced the porosity due to the increased density of TiO_2_ nanoparticles within the chitosan matrix. The high availability of TiO_2_ nanoparticles, which act as cross-linking points, leads to tighter packing of the polymer matrix, with smaller pores, and less numerous when compared with previous membranes. Higher magnification images (Figure 10c,d) indicate that many of these pores are shallow, lacking depth in the membrane structure. Therefore, this composite membrane might present a lower adsorption capacity for pollutants. The identified TiO_2_ nanoparticle agglomerates seem embedded into the chitosan matrix, and well dispersed across the membrane’s surface (Figure 10b).

**Figure 11:**
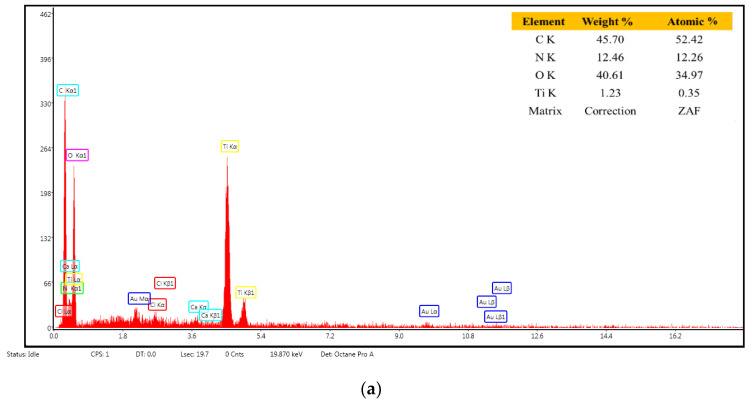

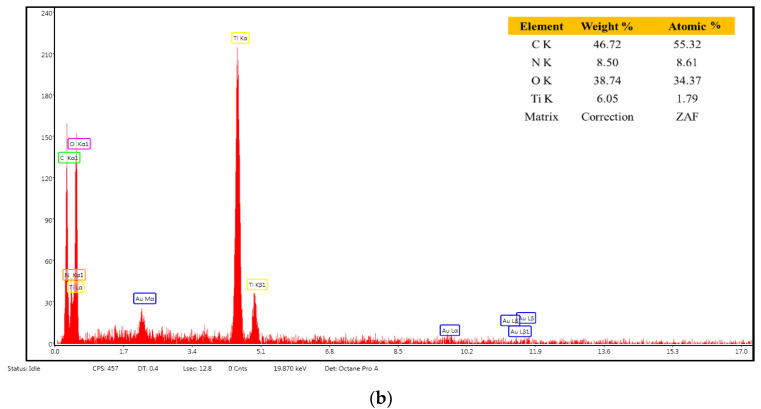
EDX spectra and elemental composition: (**a**) chitosan/TiO_2_ 1%, (**b**) chitosan/TiO_2_ 5%.

EDX analysis (Figure 11a,b) indicates the elemental composition of the tested samples. The results confirm that a higher Ti atom concentration is found in the composite membrane chitosan/TiO_2_ 5%, for both samples the percentage being close to the theoretical one.

### 3.5. Adsorption of Heavy Metal Ions (Cd and Pb)

The composite samples were immersed in cadmium or lead nitrate solutions to assess the retention capacity of each obtained composite membrane. Determinations of the Cd and Pb ion quantity adsorbed by the membranes were conducted by ICP-MS analysis. Table 3 presents the obtained results verified by a certified reference material based on heavy metal solutions.

The experimental results confirmed the SEM data and our starting hypothesis. The sample chitosan/TiO_2_ 1%, having a higher porosity and more free functional groups than the chitosan matrix, is exhibiting a higher value for the removal capacity for both Cd and Pb when compared with the sample chitosan/TiO_2_ 5%. In addition to the different membrane porosity as revealed by SEM micrographs, the higher TiO_2_ content leads to more functional groups of chitosan being involved in interactions with the nanoparticle surfaces. This diminishes the available groups that can be involved in heavy metal ion adsorption, thus confirming our starting hypothesis.

The calculated removal efficiency (1% solutions) for the chitosan/TiO_2_ 1% membrane (81.9% for Pb and 38.1% for Cd) was higher than for the chitosan/TiO_2_ 5% one (58.3% for Pb and 35.4% for Cd), due to the higher porosity as seen in SEM micrographs and higher number of free amino and hydroxyl functional groups. Table 4 presents a literature comparison with previously reported results.

The literature reports quite a few methods for regeneration of the chitosan-based membranes used, with the aim of enhancing the reusability and preserving the performance level [80,81,82]. For regeneration and reuse, depending on the nature of the adsorbate, the desorbing agent can be acid, salt, base, chelating agent, etc. For cations, usually an acidic solution is used for regeneration. By adding an acidic eluent, the amino groups in chitosan become protonated, and this favors the desorption of cations [83]. Efficient regeneration of the membrane, without major loss in the adsorption capacity, photocatalytic or antimicrobial activities, is highly desirable due to operating costs and elimination of secondary waste. Therefore, further studies are required to determine the regeneration and reusability capacity of these membranes.

### 3.6. Photocatalytic Activity Determination

**Figure 12:**
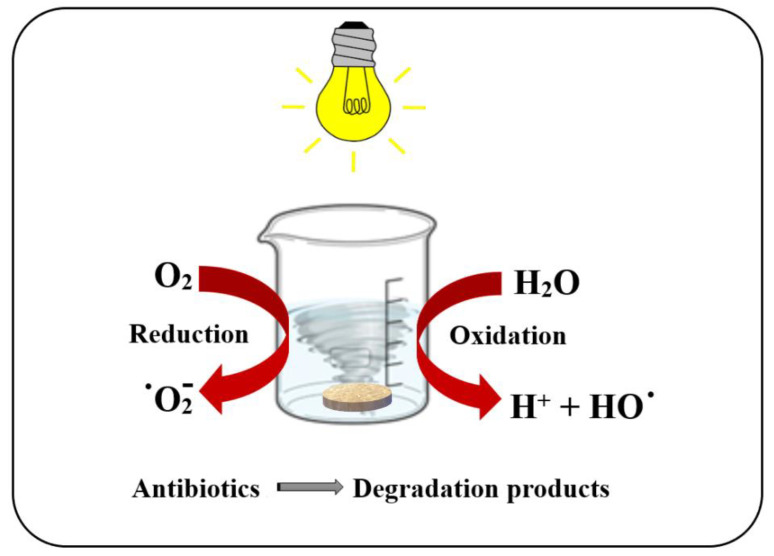
The schematic setup for the photocatalytic test.

The photocatalytic activity of the chitosan/TiO_2_ composite membranes was tested against a solution containing a mix of antibiotics (50 ppb each) by using visible light, as indicated in Figure 12.

**Figure 13:**
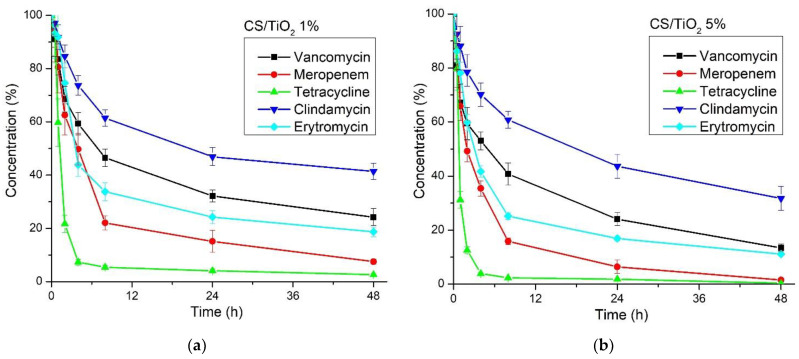
The percentile concentration of each antibiotic after irradiation in the presence of (**a**) CS/TiO_2_ 1% and (**b**) CS/TiO_2_ 5% membranes.

TiO_2_ is a well-known photocatalyst under UV light [84,85]. In this research, we have proven that it can be used in composite materials to purify the water under visible light irradiation. The results obtained after LC-MS analysis (Figure 13) indicated that both membranes have an excellent capacity to degrade the antibiotics under visible light irradiation. Nevertheless, the membrane chitosan/TiO_2_ 5% has a superior performance.

The removal efficiency values, calculated as the percent of initial antibiotic concentration removed during 48 h of irradiation, are presented in Table 5.

The best removal efficiency was obtained for tetracycline, followed by meropenem, while the lowest degradation performance was recorded for clindamycin (still ~68% for the chitosan/TiO_2_ 5% membrane).

**Figure 14:**
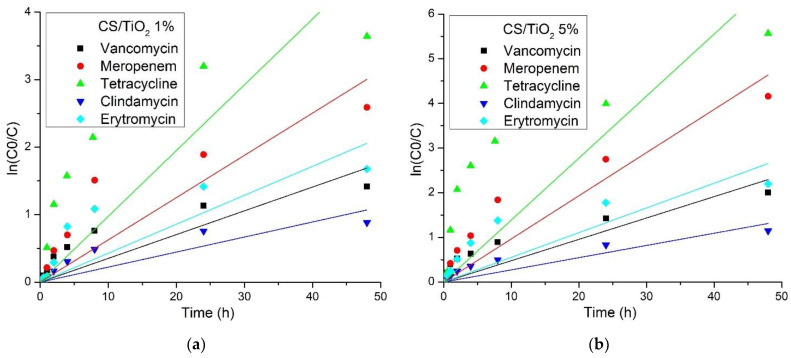
The plots of ln(C_0_/C) vs. irradiation time (t) for (**a**) CS/TiO_2_ 1% and (**b**) CS/TiO_2_ 5% membranes.

**Figure 15:**
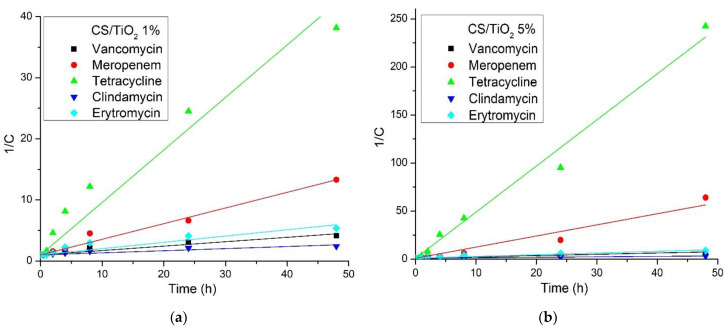
The plots of 1/C vs. irradiation time (t) for (**a**) CS/TiO_2_ 1% and (**b**) CS/TiO_2_ 5% membranes.

In order to compare our results with the previous ones reported in the literature (there are available reports on chitosan/TiO_2_ composites for tetracycline only), the rate constant must be determined. The literature reports pseudo-first- and pseudo-second-order kinetics for the photocatalytic degradation of various organics by TiO_2_ [59,86,87,88]. To establish the best fitting model, we represented graphically both types of reactions according to the following equations:

ln(C_0_/C) = k_obs1_∙t     for a pseudo-first-order kinetic and,

1/C = 1/C_0_ + k_obs2_∙t     for a pseudo-second-order kinetic,

where C_0_ and C represent initial concentration and the concentration at time t, and k_obs1_ and k_obs2_ represent the pseudo-first-order and pseudo-second-order rate constants. The plots ln(C_0_/C) and 1/C vs time (t) are presented in Figure 14 and Figure 15, respectively. Figure 14a and Figure 15a are present the plots for the chitosan/TiO_2_ 1% membrane and Figure 14b and Figure 15b present the plots for the chitosan/TiO_2_ 1% membrane.

The values calculated for k_obs1_ and k_obs2_ are presented in Table 6, together with the corresponding correlation coefficients. From the obtained results, we can see that the pseudo-second-order model gave the better fit to the experimental data, with an R_2_^2^ between 0.9003 and 0.9925.

Some studies on the photocatalytic degradation of tetracycline with chitosan/TiO_2_ composites [59] have indicated that the rate constant for a pseudo-first-order reaction is dependent on antibiotic concentration, with the best value of 0.0117 min^−1^ for 20 mg/L tetracycline concentration (20 ppm). This rate is in fact quite low due to the introduction of some heteropolyacids into the composite material; the best efficiency reported is only 66.67%. A higher removal rate of 85% under simulated sun radiation was reported in [61], against a 10 mg/L tetracycline solution (10 ppm), with a pseudo-first-order rate constant of 0.0322 min^−1^. This rate constant is still only a fraction of the ones we reported in the present study.

By using N- and S- doped TiO_2_, Farhadian et al., [89] obtained a constant rate of 0.048 min^−1^ for photocatalytic degradation of a 25 ppm tetracycline solution. In another study, [90], the authors report a pseudo-second-order rate constant of 0.0014 L∙mg^−1^∙min^−1^ for the best removal efficiency of 77% in the case of tetracycline in a photoreactor with UV irradiation and bubbling O_2_. A 97.2% removal efficiency of tetracycline from a 20 ppm solution under UV irradiation is reported in [91] by using a composite chitosan/TiO_2_ + ZnO/graphene, in this case ZnO being the main photocatalyst. The complete removal of tetracycline is reported in [92]. This was achieved under UV irradiation and in the presence of H_2_O_2_ with a composite made from alginate/chitosan/TiO_2_. Comparing the results from the literature with those reported in the present study indicates that our composite membranes exhibited a better performance, under visible light irradiation, with no added O_2_ or H_2_O_2_, with the best removal efficiency of 99.62% and rate constants with orders of magnitude higher.

For meropenem, for which we report here a removal efficiency of 98.44%, the literature is scarce. In [93], the authors report a removal efficiency of 80% under direct solar irradiation (concentrated with mirrors), using 50 mg pure TiO_2_ for a starting solution with a concentration identical with ours (only meropenem—50 ppb).

### 3.7. Swelling Study

**Figure 16:**
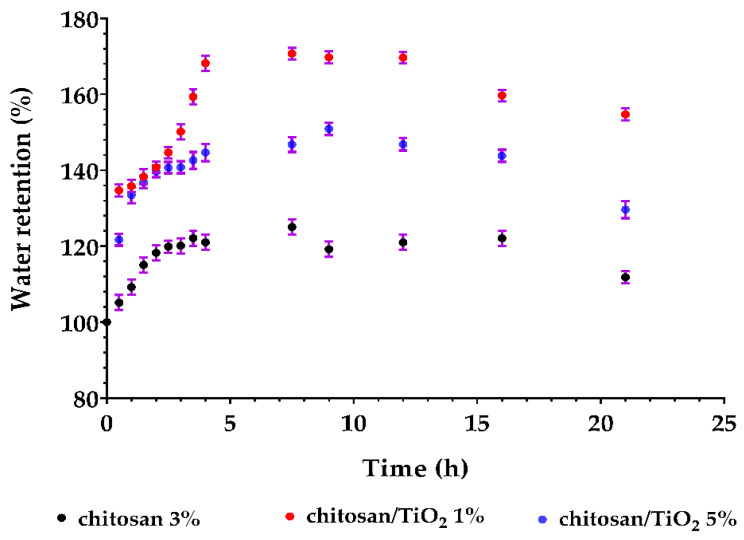
Swelling behavior for the chitosan and chitosan/TiO_2_ composite membranes.

To evaluate the sample stability, the composite membranes were subjected to swelling measurements to determine the weight change during water immersion. Figure 16 illustrates the water uptake capacity of the obtained composite membranes. It can be observed that in the first 4–6 h, the membrane mass is increasing up to a maximum value for each membrane type. After saturation is attained, the mass is relatively constant up to 8–10 h, and afterwards a slight decrease is recorded.

This behavior is caused by the partial disintegration/dissolution of the membrane surface, which will lead to some mass loss, as can be observed in the time interval 10–24 h. Similar mass loss during swelling tests are reported in the literature for other polysaccharide membranes [18,94]. The porous nature of the chitosan/TiO_2_ 1% membrane allows it a higher swelling capacity (~170%), while the chitosan/TiO_2_ 5% membrane, with fewer and smaller pores, exhibits a smaller water retention capacity (~150%).

### 3.8. The Antibacterial Assessments 

The antibacterial activity of TiO_2_ nanoparticles depends on their size, shape, morphology, crystalline structure and photocatalytic activity [95,96,97]. The size, shape and crystal structure are the most important properties that influence the physicochemical properties and antibacterial activity of TiO_2_ nanoparticles [96,97]. TiO_2_ acts on microbial cells again by affecting the cell wall and membrane by damaging DNA and decreasing/stopping the DNA replication and protein processes [98]. In another way, the cells exposed to TiO_2_ show a rapid inactivation at the regulatory and signaling levels, affecting the coenzyme-independent respiratory chains and assimilating processes of transport ions, respectively, damaging the biosynthesis of Fe-S clusters [99,100]. Moreover, the antibacterial activity of biodegradable chitosan/TiO_2_ composite membranes can also be explained by the bacteriostatic effect of the chitosan [101,102].

In this study, the antibacterial activity of the composite membranes was evaluated against Gram-negative and Gram-positive bacteria. The chitosan/TiO_2_ membranes presented bacteriostatic activity for all strains tested. The most sensitive strain on the action of TiO_2_ nanoparticles was *E. coli* ATCC 25922, followed by *Citrobacter* sp. 2021 (clinical strain). The TiO_2_ determined a sensitivity to both Gram-negative bacteria, with a decrease of at least 5 logarithmic units of CFU/mL compared to the cell growth control (C+). The chitosan presents moderate antimicrobial activity on all strains.

**Figure 17:**
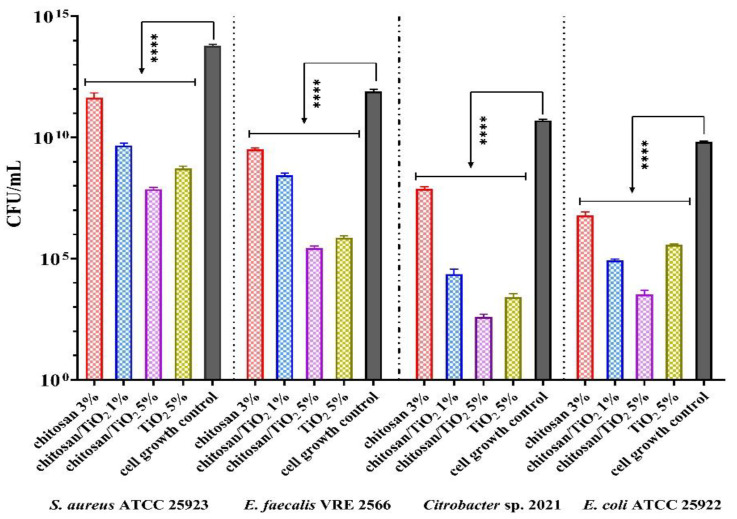
The influence of chitosan-based membranes against Gram-positive and Gram-negative bacteria. The significant differences between groups and cell wall control were statistically analyzed using one-way ANOVA, followed by Dunnett’s multiple comparisons test (**** *p* < 0.0001).

Figure 17 shows a synergic and significant antibacterial effect of both chitosan/TiO_2_ composite membranes. The antibacterial activity was enhanced due to the chitosan and TiO_2_ combination. For *Citrobacter* sp., the chitosan/TiO_2_ 1% and chitosan/TiO_2_ 5% have a pronounced high degree of inhibition (more than 8 logarithmic units of CFU/mL of C+). In addition, these samples showed a stronger antibacterial effect than TiO_2_ or chitosan. In the case of the Gram-positive bacteria, the composite membranes presented moderate antibacterial activity against both drug-resistance strains. The *E. faecalis* was more sensitive to the influence of the tested membranes.

The sensitivity of the bacterial strains can also be explained by the small size of the TiO_2_ nanoparticles (~30 nm), which have a higher interaction with the bacterial cells and activate the damage processes [96].

In another study [103], the antibacterial activity of chitosan/TiO_2_ composite film was evaluated for *E. coli*, *S. aureus*, *C. albicans* and *A. niger* strains (~10^6^ CFU/mL) under visible light. The most sensitive strain was *E coli*, with a final bactericidal ratio of approx. 100%. In addition, Siripatrawan et al. [104] reported a higher growth inhibition of the tested bacteria and fungi (*S. aureus*, *E. coli*, *Salmonella*, *P. aeruginosa*, *Aspergillus,* etc.) for the exposure of chitosan/TiO_2_ films to UV radiation than those samples without exposure. 

The lower capacity of the composite membranes to adhere to the surface determined the perspective/potential of using them in water purification applications. 

## 4. Conclusions

In conclusion, we successfully synthesized the composite membranes based on chitosan and TiO_2_ nanoparticles. These composite membranes were used to remove toxic pollutants from wastewater in a complex water purification process (adsorption of heavy metal ions, photocatalytic degradation a five-antibiotic mix and antibacterial activity against four bacterial strains). The SEM images showed TiO_2_ nanoparticle agglomerations on the surface of the membrane’s porous structure.

The heavy metal ion adsorption capacity indicated high values for Cd (315 mg/g) and Pb (297 mg/g) for the chitosan/TiO_2_ 1% membrane. This confirms the hypothesis that a decrease in TiO_2_ quantity in the composite membrane will free more of the chitosan’s functional groups, enhancing the adsorption capacity. Nevertheless, both membranes have good adsorption capacity, with the values obtained for chitosan/TiO_2_ 5% being around 255 mg/g for both Cd and Pb.

The photocatalytic activity of the membranes was determined against a mix of five antibiotic solutions under visible light irradiation. The composite membranes practically removed both tetracycline and meropenem with efficiencies over 98%, while for vancomycin and erythromycin the efficiencies were 86% and 88%, respectively.

The antibacterial assessment indicated a pronounced degree of inhibition in the case of Gram-negative bacteria due to a synergic activity of chitosan and TiO_2_, with a reduction of 8 units log CFU/mL obtained for *Citrobacter* sp. In the case of Gram-positive bacteria, the tested composite membranes presented moderate antibacterial activity against both strains.

In conclusion, it can be stated that both tested composite membranes can be used for complex water purification.

## Figures and Tables

**Table 1:** Assignment of relevant IR absorption bands of chitosan and TiO_2_.

No.	Characteristic Functional Groups	CS 3% (cm^−1^)	CS/TiO_2_ 1% (cm^−1^)	CS/TiO_2_ 5% (cm^−1^)
1	O-H stretching vibration	3352	3344	3356
	N-H stretching vibration	3182	3178	3187
2	Asymmetric stretching of Csp3-H	2924	2924	2924
	Symmetric stretching of Csp3-H	2875	2875	2875
3	Amide I band C=O stretching	1631 sh	1627 sh	1627 sh
4	Amide II band in plane N-H bending	1542	1547	1551
5	δ C-H	1404	1408	1408
6	Asymmetric stretching C-O-C	1150	1150	1150
7	C-O stretching	1065	1065	1069
		1021	1021	1021
8	Ti-O-Ti stretching vibrations	-	485	504
		-	480	504

**Table 2:** Information regarding the TG/DSC analysis of chitosan/TiO_2_ composite membranes.

Sample	Mass Loss RT-105 °C	Mass Loss 105–200 °C	Mass Loss 200–370 °C	Residual Mass at 900 °C	Endo	Exo I	Exo II
**CS 3%**	9.80%	14.67%	43.14%	2.63%	71.6 °C	293.1 °C	490.8 °C
**CS/TiO_2_ 1%**	7.73%	13.42%	40.68%	11.35%	78.8 °C	288.8 °C	498.3 °C
**CS/TiO_2_ 5%**	7.76%	11.89%	37.80%	18.00%	69.0 °C	294.0 °C	504.2 °C

**Table 3:** Cd and Pb removal capacity of chitosan/TiO_2_ composite membranes.

Sample	Metal Final Concentration	
	Pb (µg/mg)	Cd (µg/mg)
CS/TiO_2_ 1% Pb 1%	256.1 ± 3.1	
CS/TiO_2_ 1% Pb 5%	297.0 ± 4.8	
CS/TiO_2_ 1% Cd 1%		90.7 ± 1.6
CS/TiO_2_ 1% Cd 5%		315.1 ± 2.7
CS/TiO_2_ 5% Pb 1%	182.2 ± 1.9	
CS/TiO_2_ 5% Pb 5%	255.1 ± 4.2	
CS/TiO_2_ 5% Cd 1%		84.2 ± 1.5
CS/TiO_2_ 5% Cd 5%		255.0 ± 3.3

**Table 4:** Comparison with previously reported results from literature for chitosan/TiO_2_ membranes.

Composite Membrane	Removal Capacity (u.m.)	Pollutant	Reference
Chitosan/TiO_2_ (1%)	297.0 mg/g	Pb(II)	This study
	315.1 mg/g	Cd(II)	
Chitosan/TiO_2_ (5%)	255.1 mg/g	Pb(II)	This study
	255.0 mg/g	Cd(II)	
Chitosan/TiO_2_	32.1 mg/g	Pb(II)	[76]
Chitosan/TiO_2_ hybrid film	36.8 mg/g	Pb(II)	[77]
EDTA/Chitosan/TiO_2_ nanocomposite	209 mg/g	Cd(II)	[56]
Chitosan/TiO_2_ composite	256 mg/g	Cd(II)	[78]
Chitosan-Hemicellulose-TiO_2_ composite	27.6 mg/g	Cd(II)	[79]

**Table 5:** Antibiotic removal efficiency (%) of chitosan/TiO_2_ composite membranes at 48 h.

Antibiotic	Vancomycin	Meropenem	Tetracycline	Clindamycin	Erythromycin
CS/TiO_2_ 1%	75.79%	92.49%	97.38%	58.64%	81.31%
CS/TiO_2_ 5%	86.55%	98.44%	99.62%	68.26%	88.89%

**Table 6:** Values for the rate constants k_obs1_ and k_obs2_ and corresponding correlation coefficients.

Membrane Parameter/ Antibiotic	CS/TiO_2_ 1%				CS/TiO_2_ 5%			
	k_obs1_∙10^−3^ (min^−1^)	R_1_^2^	k_obs2_ (L∙mg^−1^∙min^−1^)	R_2_^2^	k_obs1_∙10^−3^ (min^−1^)	R_1_^2^	k_obs2_ (L∙mg^−1^∙min^−1^)	R_2_^2^
**Clindamycin**	0.3715	0.8366	0.0115	0.9803	0.4555	0.8866	0.0159	0.9905
**Vancomycin**	0.5872	0.8269	0.0240	0.9756	0.7967	0.8583	0.0452	0.9925
**Erythromycin**	0.7148	0.7775	0.0343	0.9499	0.9217	0.7993	0.0599	0.9708
**Meropenem**	1.0435	0.8551	0.0854	0.9887	1.6095	0.9103	0.3853	0.9337
**Tetracycline**	1.6243	0.6337	0.2863	0.9003	2.3122	0.7018	1.5982	0.9256

## Data Availability

Not applicable.
